# A critical review of Ginger’s (*Zingiber officinale*) antioxidant, anti-inflammatory, and immunomodulatory activities

**DOI:** 10.3389/fnut.2024.1364836

**Published:** 2024-06-06

**Authors:** Fitriyono Ayustaningwarno, Gemala Anjani, Azzahra Mutiara Ayu, Vincenzo Fogliano

**Affiliations:** ^1^Nutrition Science Department, Faculty of Medicine, Universitas Diponegoro, Semarang, Indonesia; ^2^Center of Nutrition Research (CENURE), Universitas Diponegoro, Semarang, Indonesia; ^3^Food Quality and Design, Wageningen University & Research, Wageningen, Netherlands

**Keywords:** ginger, immunomodulators, immune system, antioxidant, anti-inflammatory

## Abstract

Ginger (*Zingiber officinale*) is a rhizome that has been used as a healthy herbal plant for years. Ginger’s chemical components are recognized to provide beneficial health effects, namely as antioxidants and anti-inflammatory agents with the potential to operate as immunomodulators. This literature review covers numerous publications concerning ginger’s immunomodulatory potential, associated with antioxidant and anti-inflammatory effects in modifying the body’s immune system. Pathophysiology of oxidative stress and inflammation were introduced before diving deep down into the herbal plants as an immunomodulator. Ginger’s antioxidant and anti-inflammatory properties are provided by gingerol, shogaols, paradol, and zingerone. Ginger’s antioxidant mechanism is linked to Nrf2 signaling pathway activation. Its anti-inflammatory mechanism is linked to Akt inhibition and NF-KB activation, triggering the release of anti-inflammatory cytokines while reducing proinflammatory cytokines. Ginger consumption as food and drink was also explored. Overall, ginger and its active components have been shown to have strong antioxidant properties and the potential to reduce inflammation. Challenges and future prospects of ginger are also elaborated for future development. Future collaborations between researchers from various fields, including chemists, biologists, clinicians, pharmacists, and the food industry, are required further to investigate the effect of ginger on human immunity. Collaboration between researchers and industry can help accelerate the advancement of ginger applications.

## 1 Introduction

Ginger (*Zingiber officinale*), belonging to the *Zingiberaceae* family, has been widely used as a spice in various foods and beverages worldwide. In Southeast Asia, ginger has long been used as a traditional medicine to treat digestive problems, sore throats, coughs, fevers, and so on (1). The biological activity of ginger comes from the content of volatile and non-volatile compounds. The volatile components in ginger are essential oils with a distinctive aroma, which include sesquiterpenes and monoterpenoids. In contrast, the non-volatile components give ginger a pungent, spicy taste, including gingerol, shogaol, zingerone, and paradol. In fresh ginger, the main component is gingerol, which will then be converted to shogaol, zingerone, and paradol in ginger-based products (2).

The immune system is critical to the body’s physiological and immunological function. Therefore, any immune system disturbance can result in various diseases (3, 4). The immune system is a complex framework made up primarily of leukocytes and various immune components such as antibodies, proteins, and cytokines that serve as the first line of defense against a wide range of potentially harmful substances in the environment. The interaction of these different immune components makes it easier to establish an optimal immune response (5). In order to prevent various disorders, the immune system identifies and eliminates pathogens while inducing inflammation, cell or tissue damage, cell death, and wound healing. Immune-mediated diseases, metabolic disorders, and inflammatory diseases can occur if homeostasis is not maintained, as well as susceptibility to infectious diseases such as COVID-19 (6, 7).

Degenerative diseases, such as diabetes mellitus and hypertension, cause high mortality rates. Degenerative diseases also increase the severity of infectious diseases. Oxidative stress due to free radicals from the environment is still a threat during a pandemic, so it can disrupt the immune system. Therefore, the easiest and most immediate way to prevent or reduce the transmission of infectious diseases is through clean and healthy living behaviors, social restrictions, and increasing body immunity (8).

The body’s immune function is essential for prevention, disease recovery, and reducing the risk of infectious and chronic disease. Increasing the body’s immunity can be done by eating balanced nutritious foods supplemented by vitamins, minerals, and bioactive compounds. Herbal plants contain bioactive compounds that can boost the immune system. In addition, herbal plants can also provide therapeutic benefits and have the potential to have antioxidant activity that can suppress free radicals and prevent oxidative stress and inflammation in the body (8).

Immunomodulators are substances that help to regulate the immune system (9). Immunomodulators are available in both endogenous and exogenous forms, with the mechanism often based on antioxidant properties. Some natural plant antioxidants have been shown to have immunomodulatory properties including alkaloids, flavonoids, flavones, flavonols, flavanols, isoflavones, quinones, terpenoid (10). Immunomodulators derived from antioxidants help maintain health by regulating the oxidant-antioxidant balance and immune response. Antioxidants work both directly and indirectly by eliminating reactive oxygen-nitrogen species and increasing the body’s antioxidant balance (11). Many plants’ bioactive compounds have been investigated for their potential benefits as immunomodulators (9).

Various studies have explored ginger’s biological activity, the active components that contribute, and their mechanism of action (12). *In vitro* and *in vivo* research demonstrate that ginger crude extract has biological activities such as antioxidant, anti-inflammatory, antibacterial, antiviral, and anticancer properties (1). Some recent papers showed the different aspects of ginger. The therapeutic role of ginger has been studied by Kausar et al., including antioxidant effect, anti-nausea effect, anti-inflammatory effect, anti-cancer effect, cardiovascular effect, and anti-diabetic effect (13).

The recent review by Mao (1), discussed the bioactive compound and bioactivities of ginger, including antioxidant activity, anti-inflammatory activity, antimicrobial activity, neuroprotection, anti-obesity activity, anti-diabetic activity, protective effect against respiratory disorders, also the other bioactivities of ginger (hepatoprotective and antiallergic effects) (1). Jalali (14) in a systematic review and meta-analysis, describe ginger supplementation’s effect on inflammatory and oxidative stress markers. A recent study by Ghafoor (15) shows the effect of drying methods on the total phenolic and antioxidant activity. Morvaridzadeh (16) in a systematic review and meta-analysis, studied the effect of ginger supplementation on oxidative stress parameters.

Based on this existing background information, this review will consider the antioxidant and anti-inflammatory role of ginger in the immunomodulatory system since it is a relevant but underexposed factor.

## 2 Method

Scientific information on Ginger’s (*Zingiber officinale*) Antioxidant, Anti-Inflammatory and Immunomodulatory Activities and their bioactive compounds were gathered. Keywords used including *Zingiber officinale* antioxidant, *Zingiber officinale* anti-inflamatory, *Zingiber officinale* Immunomodulatory. A literature search was conducted using Google Scholar. Published scientific papers from respected publisher such as ACS, Frontiers, ScienceDirect, Springer, Emerald, and others was used.

## 3 Discussion

### 3.1 Ginger (*Zingiber officinale*)

Ginger (*Zingiber officinale*) has been widely used as a spice in various foods and beverages around the world. In Southeast Asia in particular, ginger has also long been used as a traditional medicine to treat digestive problems, sore throats, coughs, fevers, and so on (1). While in China, it is known as an herbal plant that is widely used in traditional medicine (17). In Indonesia, ginger has long been known and used as a spice, medicinal plant, as well as flavor and aroma in various foods and beverages. Ginger is a rhizome plant that has been developed in the medicinal industry as perfume and traditional herbal medicine. Young ginger is eaten as fresh vegetables, processed into pickles, and used as an ingredient in beverages (18).

Ginger is a seasonal herbaceous plant, pseudo-trunked and erect with a height of between 30 cm to 1 m, leaf length of 15–23 mm, leaf width of 8–15 mm, and 2–4 mm long hairy petioles. Morphologically, the ginger plant consists of roots, rhizomes, stems, leaves, and flowers. Ginger has rhizome roots that can last a long time in the soil and can produce new shoots to replace dead leaves and stems. Ginger rhizome branched irregularly, coarsely fibrous, spreading flat and pale-yellow inside. Roots grow from the bottom of the rhizome while shoots will grow from the top of the rhizome (18). In fact, ginger has different shapes and flavors based on its type (Table 1).

**Table 1:** Comparison of the morphology and content between different ginger’s variety.

Characteristics	Elephant Ginger (*Zingiber officinale* var. *officinarum*)	Emprit Ginger (*Zingiber officinale* var. *amarum*)	Red ginger (*Zingiber officinale* var. *rubrum*)	Reference
Diameter	48–85 mm	32.7–40 mm	42–43 mm	*
Tall	62–113 mm	63.8–111 mm	52–104 mm	*
Long	158–327 mm	61–317 mm	123–126 mm	*
Weight Per Clump	1–2 kg	0.5–0.7 kg	0.5–0.7 kg	*
Shape	Big and fat, soft fiber	Small and layered, coarse fiber	Small and layered, coarse fiber	*
Rhizome Color	Yellowish white	Yellowish white	Light orange-red	*
Ginger’s Spiciness	Less spicy	Spicy	Very spicy	*
Ginger’s Aromatic	Less strong	Strong	Very strong	*
Essential oil (%)	1.62–2.29	3.05–3.45	3.90	**
Starch (%)	55.10	54.70	44.99	**
Fiber (%)	6.89	6.59	8.99	**

Reference* (18) and ** (19).

Ginger has a lot of active compounds such as phenolics and terpenoids. Ginger contains phenolic elements such as gingerol, shogaol, zingerone, and paradol. The major polyphenols in fresh ginger are gingerols. Ginger also contains a variety of phenolic chemicals and terpenoid components, which are regarded to be the major ingredients of ginger essential oil. Ginger also contains carbohydrates, lipids, organic acids, fiber, and other nutrients (Table 2) (1).

**Table 2:** Nutrition content of ginger.

Nutritional and bioactive content	Amount Per 100 g	Reference
Moisture	8.8	*
Protein	9.01	*
Fat	1.33	*
Fiber	6.62	*
Ash	6.33	*
Carbohydrate	67.81	*
Energy (Kcal)	319.74	*
Macro minerals (g/kg)		**
P	3.81	**
K	33.84	**
Na	0.71	**
Ca	1.80	**
Mg	2.24	**
Micro minerals (g/kg)		**
Fe	0.230	**
Zn	0.037	**
Mn	0.068	**
Phenolic		
Gingerol (%)	23–35	***
Shogaol (%)	18–25	***
Terpenoids		
Curcumene (%)	4.71	****
Camphene (%)	3.02	****
Zingiberene (%)	39.01	****
Zingeberon (%)	1.20	****

Reference * (20), ** (21), *** (22), and **** (23).

#### 3.1.1 Elephant ginger (*Zingiber officinale* var. *officinarum*)

**Figure 1:**
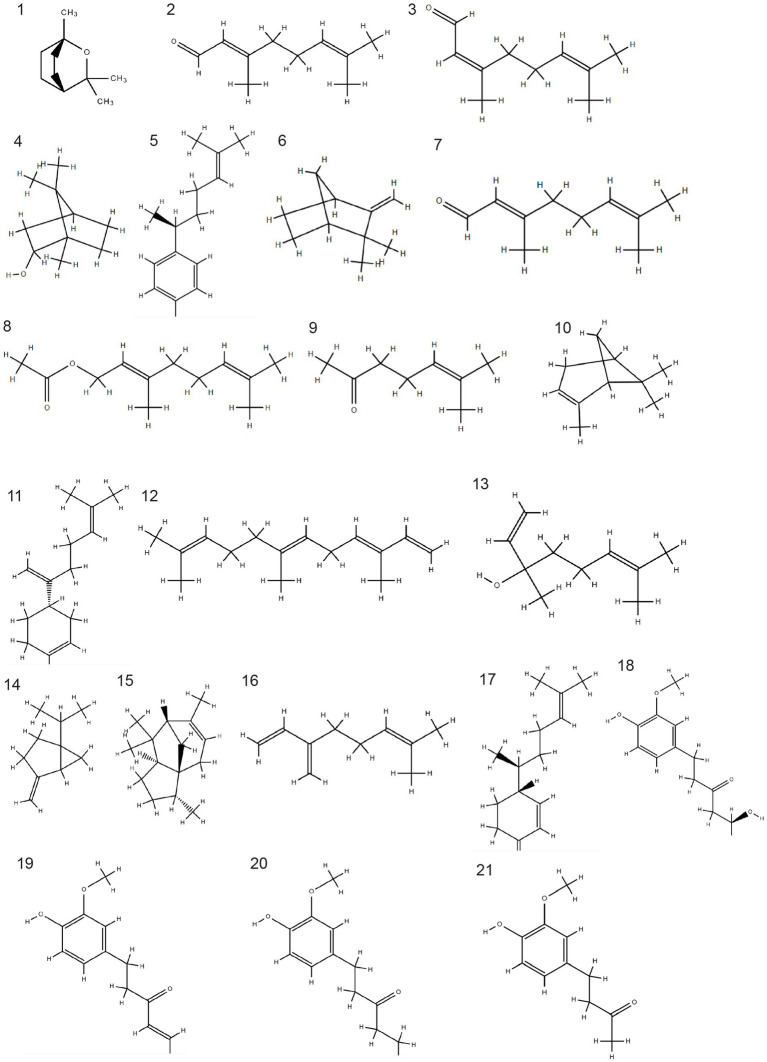
Chemical structure of bioactive components in three varieties of ginger. 1. 1,8-cineole, 2. geranial, 3. neral, 4. borneol, 5 ar-curcumene, 6. camphene, 7. citral, 8. geranyl acetate, 9. methyl heptanone, 10. α-pinene, 11. Β-bisabolene, 12. fernesene, 13. linalool, 14. sabinene, 15. α-cedrene, 16. β-myrcene, 17. β-sesquiphellandrene, 18. gingerol, 19. shogaol, 20. paradol, 21. zingerone. The SDF coordinate generated by PubChem and the chemical structure was build using Chemspider.

Elephant ginger is commonly utilized in culinary applications and herbal beverages due to its large rhizome, a milder odor and a relatively lower fiber content and concentration of essential oil compared to other varieties of ginger. Its essential oil comprises 45 components, including 1,8-cineole (6.4%), ar-curcumene (2.75%), camphene (6.48%), citral (16.19%), fernesene (3.8%), linalool (2.57%), methylheptenone (2.33%), sabinene (6.19%), z-citral (neral) (11.84%), α-cedrene (4.72%), α-pinene (3.22%), β-bisabolene (2.28%), β-myrcene (2.48%), and β-sesquiphellandrene (3.35%). Chemical structure of each components can be observed in Figure 1. A previous study revealed that elephant ginger infusion extraction yielded the lowest phenolic content (7.6400 ± 0.21 mgGAE/g) and exhibited the least antioxidant activity (61.7000 ± 1.51) based on the DPPH method when compared with two other ginger variations (24, 25).

#### 3.1.2 Emprit ginger (*Zingiber officinale* var. *amarum*)

Emprit ginger is also utilized for culinary and herbal applications albeit with distinct characteristics compared to elephant ginger. Unlike elephant ginger, emprit ginger features a smaller rhizome, beige skin color, sharper odor, and higher fiber content. Notably, emprit ginger boasts a greater concentration of essential oil than elephant ginger. Its essential oil profile comprises 38 chemical constituents, including notable components such as 1,8-cineole (7.95%), 1,8-p-methandiene (2.74%), ar-curcumene (6.86%), Bornyl acetate (3.21%), camphene (10.14%), citral (16.19%), geranyl acetate (2.24%), methylheptenone (2.44%), z-citral (12.23%), α-pinene (2.5%), and β-bisabolene (2.64%). Chemical structure of each components can be observed in Figure 1. A preceding study observed that emprit ginger infusion extraction exhibited higher phenolic content (9.5033 ± 0.35 mgGAE/g) and displayed greater antioxidant activity (70.4333 ± 0.25) based on the DPPH method compared to elephant ginger (24, 25).

#### 3.1.3 Red ginger (*Zingiber officinale* var. *rubrum*)

Red ginger is generally utilized for dietary supplementation and traditional remedies, characterized by its relatively smaller rhizome size, red skin color, sharp aroma, and higher fiber content, as well as boasting the highest concentration of essential oil among ginger varieties. The essential oil derived from red ginger notably comprises a significant percentage of monoterpenoids (60.6%), primarily including 1.8-cineole (15.1%), geranial (12%), neral (7.1%), and borneol (6.8%). Sesquiterpene constituents are present in lower proportions compared to monoterpenes, with ar-curcumene (16.9%), β-sesquiphellandrene (6.8%), and β-bisabolene (6.4%) being the dominant components. Chemical structure of each components can be observed in Figure 1.

Previous research has indicated that red ginger exhibits the highest phenolic content in ginger infusion extraction methods (12.2533 ± 0.13 mgGAE/g) (24). Similarly, according to the DPPH method, red ginger demonstrates the highest antioxidant activity in ginger infusion extraction methods (70.4333 ± 0.25%) among the three ginger variations studied (24, 25). Additionally, Table 3 provides a detailed comparison of the chemical constituents present in the three ginger varieties.

**Table 3:** Bioactive constituents in three ginger varieties.

Chemical constituent	Elephant ginger (%)*	Emprit ginger (%)*	Red ginger (%)**
(−)-Spathulenol	–	0.44	–
1,8-Cineole	6.4	7.95	15.1
1,8-p-Menthadiene	–	2.74	–
2-Heptanone	0.21	0.19	–
2-Nonanone	0.95	1.21	–
2-Undecanone	1.84	1.54	–
4-Terpineol	0.88	–	–
4α-Methyl-trans-2-decalinone	–	0.46	–
ar-Curcumene	2.75	6.86	16.9
Bornyl acetate	0.63	3.21	–
Bornyl methyl ether	–	0.18	–
Camphene	6.48	10.14	–
Camphor	0.45	1.31	–
Caryopilen	–	0.42	–
Citral	16.19	16.19	–
Citronella	1.31	0.47	–
Citronellyl acetate	0.27	0.51	–
Cryptone	0.4	0.5	–
Cyclohexane	–	1.27	–
Cycloisolongifolene	0.31	–	–
d-3-Carene	0.26	–	–
d-Nerolidol	–	–	–
Farnesene	3.8	–	3.6
ɣ-Cadinene	–	–	–
Geranic acid	–	–	–
Geraniol formate	–	–	–
Geranyl acetate	0.68	–	–
Germacrene	0.35	–	–
ɣ-Maaliene	0.95	–	–
Isoborneol	0.58	–	6.8
Isogeraniol	–	0.55	–
Linalool	2.57	–	2.0
Methylheptenone	2.33	2.44	–
Myrtenal	–	–	–
n-Decanal	2.2	–	–
Neoallocimene	0.28	–	–
p-Cymene	0.29	0.56	–
Piperitenone	0.32	–	–
Piperitone	–	0.25	–
Sabinene	6.19	–	–
Squalene	–	0.27	–
Sylvestrene	–	–	–
Thiogeraniol	0.31	0.36	–
Tricyclene	0.32	0.4	–
Valencene	0.32	–	–
Z-Citral	11.84	12.23	7.1
Zingiberene	0.07	0.22	2.3
α-Bergamotene	0.28	–	–
α-Cedrene	4.72	–	–
α-Cubebene	0.21	–	–
α-Guaiene	0.24	–	–
α-Phellandrene	0.53	–	–
α-pinene	3.22	–	–
α-Terpineol	2.24	–	–
α-Terpinolene	0.64	–	–
β-Bisabolene	2.28	2.64	6.4
β-Elemene	0.5	0.21	–
β-Myrcene	2.48	1.09	4.0
β-Pinene	0.87	0.49	0.9
β-Sesquiphellandrene	3.35	0.4	6.8
Limonene	–	–	4.1
6-Hepten-3-one	–	–	3.2
trans-Caryophyllene	–	–	2.2
Naphtalene	–	–	1.4
Neryl acetate	–	–	1.6
Geraniol	–	–	3.9
Geranial/e-citral	–	–	12.0

Reference * (26) and ** (27).

### 3.2 Immunomodulators

The immune system works as the body’s defense against exposure to foreign substances by recognizing and killing these foreign substances. The immune system is divided into innate and adaptive immune systems, which determine the ability to fight off harmful agents. Innate immunity, which includes epithelial protection, antimicrobial proteins, peptides, humoral and cellular components is recognized as the first line of defense against pathogens. Leukocytes produced ROS to destroy the engulfed pathogen (28). The innate immune system activates the adaptive immune system, which aids in foreign antigen recognition (29). The adaptive immune responses consist of B lymphocytes with antigens, T lymphocytes, and regulatory T cells (T-reg) (30).

The most important cytokines in the body are T-lymphocytes. These cells possess antigen receptors on their cell surfaces that allow them to recognize invading pathogens. T cells are divided into CD4 and CD8. Helper T cells are T lymphocytes that express CD4. This subgroup is further separated into T helper 1 (Th1) and T helper 2 (Th2) cells, referred to as Th1-type cytokines and Th2-type cytokines. Th1-type cytokines are responsible for destroying intracellular parasites by triggering a pro-inflammatory response. Interferon (IFN-γ), tumor necrosis factor (TNF-α), interleukin-1 (IL-1), and IL-18 are examples of Th1 cytokines (31). Th2-type cytokines, on the other hand, have a more complicated mode of action. Th2 cytokines include IL-4, IL-5, IL-9, and IL-13, all of which are linked to the immunoglobulin response; IL-6, which is pro-inflammatory; and IL-10, which is anti-inflammatory. As a result, a balance of Th1 and Th2 responses is required to fight pathogens effectively while preventing uncontrolled inflammation (32). Activation of the Nuclear Factor Kappa B (NF-κB) and COX 2 signaling pathways can also increase the concentration of pro-inflammatory cytokines and the activator cascade to activate other cytokines that induce inflammation (33).

Immunity can be maintained and enhanced by consuming vitamins, minerals, and active compounds from nature that are effective immunomodulators. Antioxidant and anti-inflammatory properties may contribute and play a role as an immunomodulator. Antioxidants neutralize free radicals and prevent oxidative stress, ultimately maintaining the immune system. Anti-inflammatory is also linked with antioxidants to regulate free radical production as well as regulate cytokine pro-inflammation to prevent inflammation. The pathophysiology of oxidative stress and inflammation is essential in understanding the role of antioxidants and anti-inflammatories as immunomodulators.

#### 3.2.1 Pathophysiology of oxidative stress

Oxidative stress can be induced by free radical production. Free radicals are compounds that contain one or more unpaired electrons that are highly reactive and able to oxidize lipids, proteins, carbohydrates, and DNA (34). Free radicals can be reactive oxygen or nitrogen species (ROS/RNS), referred to as Reactive Oxygen-Nitrogen Species (RONS) (35). All aerobic cells can generate RONS as a by-product of oxidation and cell metabolic processes during respiration, cell reproduction, maximum physical activity, inflammation (inflammation), and exposure to external contaminants. Superoxide radicals (O_2_−), hydroxyl radicals (OH−), peroxyl radicals (ROO−), hydrogen peroxide radicals (H_2_O_2_), singlet oxygen radicals (½O_2_), nitric oxide radicals (NO−), peroxynitrite radicals (ONOO−), hypochlorous acid radicals (HOCl), and fat oxidation products are all reaction product of RONS (35, 36).

The major contributor of superoxide radicals (O_2_−) is NADPH oxidase, which is synthesized during cellular respiration by reducing one-electron oxygen molecules with electrons delivered by NADPH. Most O_2_ radicals were transformed into hydrogen peroxide (H_2_O_2_) radicals by Superoxide Dismutase (SOD). Because it lacks unpaired electrons, H_2_O_2_ radicals are not free radicals but can become hydroxyl ions (OH^−^), which via the Fenton or Haber-Weiss reaction, are highly reactive radicals. The hydroxyl radical (OH−) is very reactive, particularly with phospholipids in cell membranes and proteins. In neutrophils, H_2_O_2_ may combine with chloride and MPO to form hypochlorous acid radicals, the primary ROS that damage cellular proteins. L-arginine can stimulate three major isoforms of NO synthase: epithelial NO synthase, which is involved in vasodilation and vascular regulation; neuron NOS, which is involved in intracellular signaling; and receptor NOS, which is stimulated in response to various endotoxin or cytokine signal activations. Finally, O_2_ radicals can interact with NO radicals to synthesize reactive substances such as peroxynitrite radicals (ONOO−) (35, 36).

Exogenous RONS contributors include air and water pollution, tobacco, alcohol, heavy or transition metals, drugs (e.g., cyclosporine, tacrolimus, gentamicin, and bleomycin), industrial solvents, unhealthy food (e.g., smoked meats, used oils, and fats), radiation, and bacterial, fungal, and viral infections (35, 36).

#### 3.2.2 Pathophysiology of inflammation

Inflammation plays a vital role in the body’s response to a disturbance or infection. The inflammatory response is regulated by mediators released by cells around the site of infection and the circulatory system (immune cells). Mediators, including cytokines, proteins, and inflammatory enzymes, can indicate inflammation-related diseases. The various cytokines have their respective sources and roles in inflammation (Table 4).

**Table 4:** Cytokines’ various, sources, and their roles in inflammation.

Cytokines	Source	Role
TNF-α	Macrophages, NK cells, lymphocytes, and adipocytes	Pro-inflammation, cell proliferation and apoptosis
IFN-γ	T cells, NK cells, and NK cells	Antiviral and pro-inflammatory
IL-1β	Macrophages and monocytes	Pro-inflammation, proliferation, apoptosis, and cell differentiation
IL-6	Macrophages, T cells, and adipocytes	Proinflammatory and -cell differentiation
IL-8	Macrophages, epithelial, and endothelial cells	Proinflammatory, chemotaxis, and angiogenesis
IL-12	Macrophages, dendritic cells and neutrophils	Proinflammation, cell differentiation, and NK cell activation

Reference [37].

**Figure 2:**
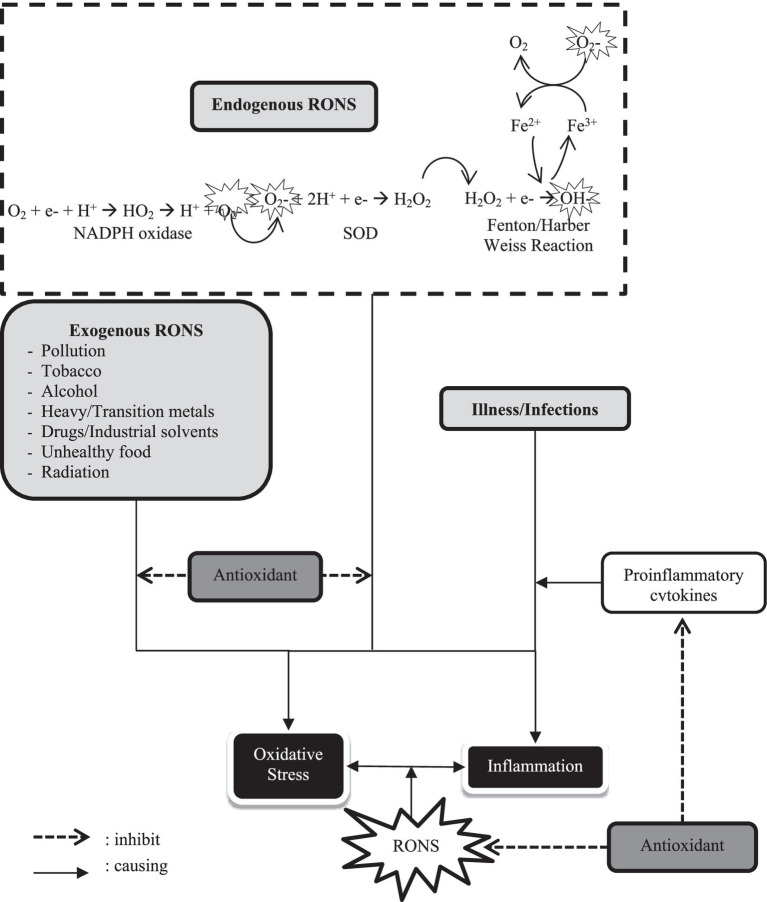
Oxidative-inflammatory stress relationship and the role of antioxidants. (O_2_−, superoxide radical; SOD, superoxide dismutase; H_2_O_2_, hydrogen peroxide; OH−, hydroxyl radical; RONS, reactive oxygen-nitrogen species).

Severe infectious disease can trigger uncontrolled cytokine production, called a cytokine storm. The excessive number of immune cells at the site of infection causes cell and tissue damage, organ failure, and even death (38). In addition, oxidative stress is also closely related to inflammation and is bidirectional. The formation of RONS triggers inflammation, but on the other hand, inflammation also triggers the formation of RONS, which ends in oxidative stress, as described in Figure 2 (33). Therefore, antioxidants play a crucial role in preventing inflammation and preventing or suppressing the onset of oxidative stress and cell tissue damage. Antioxidants can limit the production of proinflammatory cytokines such as interleukin-6 (IL-6) and tumor necrosis factor (TNF-α) (36). In addition, antioxidants increase the effectiveness of white blood cells maintaining the immune system and increasing the body’s resistance to diseases.

Respiration and metabolism produce O_2_− which is neutralized by SOD into H_2_O_2_. It can become OH− through Fenton/Harber Weiss Reaction. All those mechanisms belong to endogenous RONS production. Exogenous RONS, including pollution, tobacco, metals, drugs, etc., with endogenous RONS might be causing oxidative stress. Illness/infection can trigger inflammation by proinflammatory cytokines increasing. RONS play a role in bidirectional causes both stress oxidative and inflammatory. Antioxidants can inhibit exogenous and endogenous RONS as well as regulate proinflammatory cytokines, so it has potential as an immunomodulator.

### 3.3 Herbal plants as immunomodulator

Several endogenous and exogenous factors contribute to immunosuppression and immunostimulation during the homeostatic state, influencing innate and adaptive immune responses (10). Immune response modulation regulates immune response in therapeutic and preventive efforts against causative agents (39, 40). immunomodulators use is currently one of the most pressing issues in the treatment of various diseases.

The use of plant-derived bioactive compounds has become one of the most active areas of research for natural immunomodulators (41). Natural immunomodulators are becoming popular due to their broad application in disease prevention and treatment via changes in immune response or oxidant-antioxidant status (42).

**Figure 3:**
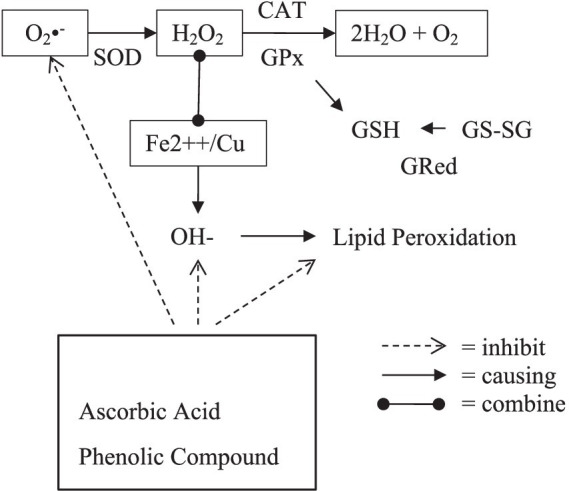
Mechanism of endogenous and exogenous antioxidant. (O_2_•^−^, superoxide radical ion; H_2_O_2_, hydrogen peroxide; OH−, hydroxyl radical; SOD, superoxide dismutase; GPx, glutathione peroxidase; GSH, glutathione; GS-SG, glutathione disulfide; GRed, glutathione reductase).

Antioxidants function as a defense in protecting the body’s biological systems from the toxicity of free radicals. Antioxidants have two different mechanisms: endogenous and exogenous, as described in Figure 3.

The body may synthesize its antioxidants (endogenous antioxidants) in biological systems, both enzymatic and non-enzymatic routes. Superoxide Dismutase (SOD), Catalase (CAT), and Glutathione Peroxidase (GPx) are forms of enzymatic antioxidants (35, 36).

SOD enzymes in the cytosol and mitochondria convert superoxide radicals (O_2_−) into hydrogen peroxide (H_2_O_2_) catalyzed by the enzymes Catalase (CAT) and Glutathione Peroxidase (GPx). The CAT enzyme uses one H_2_O_2_ molecule as an electron donor substrate and one H_2_O_2_ molecule as an electron acceptor substrate so that 2H_2_O_2_ molecules are neutralized into 2H_2_O and O_2,_ thereby preventing the production of hydroxyl radicals. In erythrocytes and other tissues, the GPx enzyme catalyzes the destruction of H_2_O_2_ and lipid hydroperoxides using reduced glutathione (GSH), protecting membrane lipids and hemoglobin from H_2_O_2_ oxidation, and preventing hemolysis caused by hydrogen peroxide attack. GSH will be oxidized to Glutathione Disulfide (GS-SG). In order for GSH to continue to be available to help the GPx enzyme work, this GS-SG must be reduced again to GSH. This function is played by the enzyme Glutathione Reductase (GRed). H_2_O_2,_ which is not converted to H_2_O, can form reactive hydroxyl radicals (OH) when reacted with transition metal ions (Fe^2++^/Cu). These hydroxyl radicals are more reactive and dangerous because they can cause cell damage through the peroxidation of lipids, proteins, and DNA.

On the other hand, the body does not have enzymes to convert hydroxyl radicals into safe molecules for cells. These hydroxyl radicals are more reactive and dangerous because they can cause cell damage through the peroxidation of lipids, proteins, and DNA. Glutathione-S-transferase and glucose-6-phosphate dehydrogenase are two different other antioxidant enzymes. Non-enzymatic antioxidants interact with RONS and stop the chain reactions of free radicals in the blood, such as tocopherols and carotenoids (35).

Increased pollution causes excessive ROS production, so endogenous antioxidants are not enough to neutralize free radicals. Therefore, the body needs antioxidants from daily food and drinks (36). Exogenous antioxidants are antioxidants that come from outside the body and are ingested, such as ascorbic acid (vitamin C), which scavenges hydroxyl and superoxide radical anions; tocopherols (vitamin E), which fight cell membrane lipid peroxidation; and phenolic antioxidants, which include stilbene derivatives (resveratrol, phenolic acids, and flavonoids), oil lecithin, selenium, and zinc (35).

During exogenous antioxidant intake deficiency, the body has the potential to experience an imbalance between the number of free radicals and the amount of antioxidants called oxidative stress. In the long term, this condition can cause cell damage that can lead to various diseases such as cancer, heart disease, cataracts, premature aging, and other degenerative diseases (36).

The antioxidant defense system consists of free radical reduction, antioxidant enzyme activation and maintenance, enhancement of other antioxidant mechanisms, and specific immunomodulatory effects (42). Plants that act as natural immunomodulators can boost immunity by activating innate immune responses such as activating immune cellular components, changing cytokine profiles, and reducing infection and inflammation. This plant’s immunomodulatory properties are linked to its bioactive compounds (10).

Herbal plants are plants or medicinal plants that can be used for traditional treatment of diseases. The use of herbal plants has been empirically proven and used to maintain a healthy body. In addition, herbal plants are also used by the community in cooking, such as the use of spices. Herbal plants are known to contain many secondary metabolites such as flavonoids, alkaloids, tannins, and triterpenoids (17). Flavonoids, for example, are phenolic chemical derivatives that can bind RONS, restrict the operation of enzymes that make RONS, and form chelates with metals that stimulate the synthesis of RONS, preventing RONS interactions with normal cells such as lipid peroxidation and DNA damage. Herbal plants are found in the form of roots, rhizomes, tubers, bark, stems, leaves, flowers, fruits, and seeds, and are frequently utilized by the community, such as curcuma, cinnamon, bay leaf, etc. Ginger rhizome is a form of rhizome that has been trusted and utilized for years. Its effectiveness is said to be as a carminative (trigger flatulence) to relieve cold symptoms, and improve appetite, and has herbal benefits to cure coughs, heartburn, dizziness, nausea, and influenza.

### 3.4 Ginger as immunomodulators

Overproduction of free radicals (RONS) plays an important role in the development of various diseases and triggers inflammation. Several studies have found that ginger has been shown to have potential as a natural immunomodulator through both antioxidant and anti-inflammation properties, or by their modulation to other immune components (Table 5), so that it can prevent oxidative stress and inflammation (1). It is known that ginger contains antioxidants with an IC50 value of 4.25 g/mL. This means that ginger has a good antioxidant effect (IC50 < 5 mg/mL). Therefore, this ginger rhizome is one of the herbal plants that is widely used by the community (17).

**Table 5:** Ginger’s antioxidant and anti-inflammatory immunomodulators.

Intervention form	Study type	Subject	Dose	Results	Reference
Ginger Extract (ethanol)	*In vitro*	HepG2 cells	100 μg/mL	Inhibits ROS production, DNA damage, and cell death	(43)
	*In vivo*	Male wistar	250 mg/kg	Increases antioxidant enzyme activity and regulates the Nrf2/HO-1 pathway	
Ginger Extract (water)	*In vivo*	Wistar albino male	300 mg/kg	Prevent oxidative stress, and improve the expression of COX-2, iNOS, and NF-κB p65	(44)
Ginger Extract (dimethyl sulfoxide)	*In vitro*	Chondrocytes C28I2	25 μg/mL	Increases gene expression on antioxidant enzymes, inhibits ROS production, lipid peroxidation, Bax/Bcl-2 ratio, and caspase-3 activity	(45)
Ginger Extract (water)	*In vivo*	Wistar albino male	24 mg/mL	Reduces cell damage by increasing T3, T4, and antioxidant enzymes (SOD, CAT), lowering MDA	(46)
Ginger Extract (ethanol)	*In vivo*	Wistar albino male	800 mg/kg	Decreased levels of MDA and protein carbonyl, increased levels of GSH, SOD, and CAT activity, decreased levels of TNF-α, IL-1β IL-6, caspase-3, and cytochrome c	(47)
Ginger Extract (water)	*In vivo*	Albino male	216 mg/kg	Lowers IL-1β, TNF-α, MDA, and NOx levels, increases GSH	(48)
Ginger Extract (water)	*In vivo*	C57BL6/J mice	50 mg/mL	Inhibits TNF-α production	(49)
Ginger Extract (water)	*In vitro*	RBC	500 μg/mL	Inhibits proteinase and anti-lipoxygenase activity	(50)
	*In vivo*	Sprague dawley male	200 mg/kg	Decreased PGE2, TNF-α, IL-6, MCP-1, and MPO. Increase antioxidant activity	
Ginger extract (ethanol)	*In vivo*	BALB/c female mice	100 mg/kg	Inhibits NF-κB activity and lowers IL-1β levels	(51)
Ginger oleoresin	*In vitro*	hMSCs	1–4 g/mL	Prevents cell damage through inhibition of ROS production, triggers Nrf2 translocation, activates HO-1 and NQO1 gene expression	(52)
Ginger Juice	*In vivo*	Male wistar	5 mL/kg	Reduced expression of iNOS and NF-κB as well as levels of NO, TNF-α, IL-6, and IL-1β	(53)
Ginger Tea	*Ex vivo*	Chicken liver tissue	100 mL 2.5%	Inhibits fat peroxidation, and suppresses free radicals	(54)

**Figure 4:**
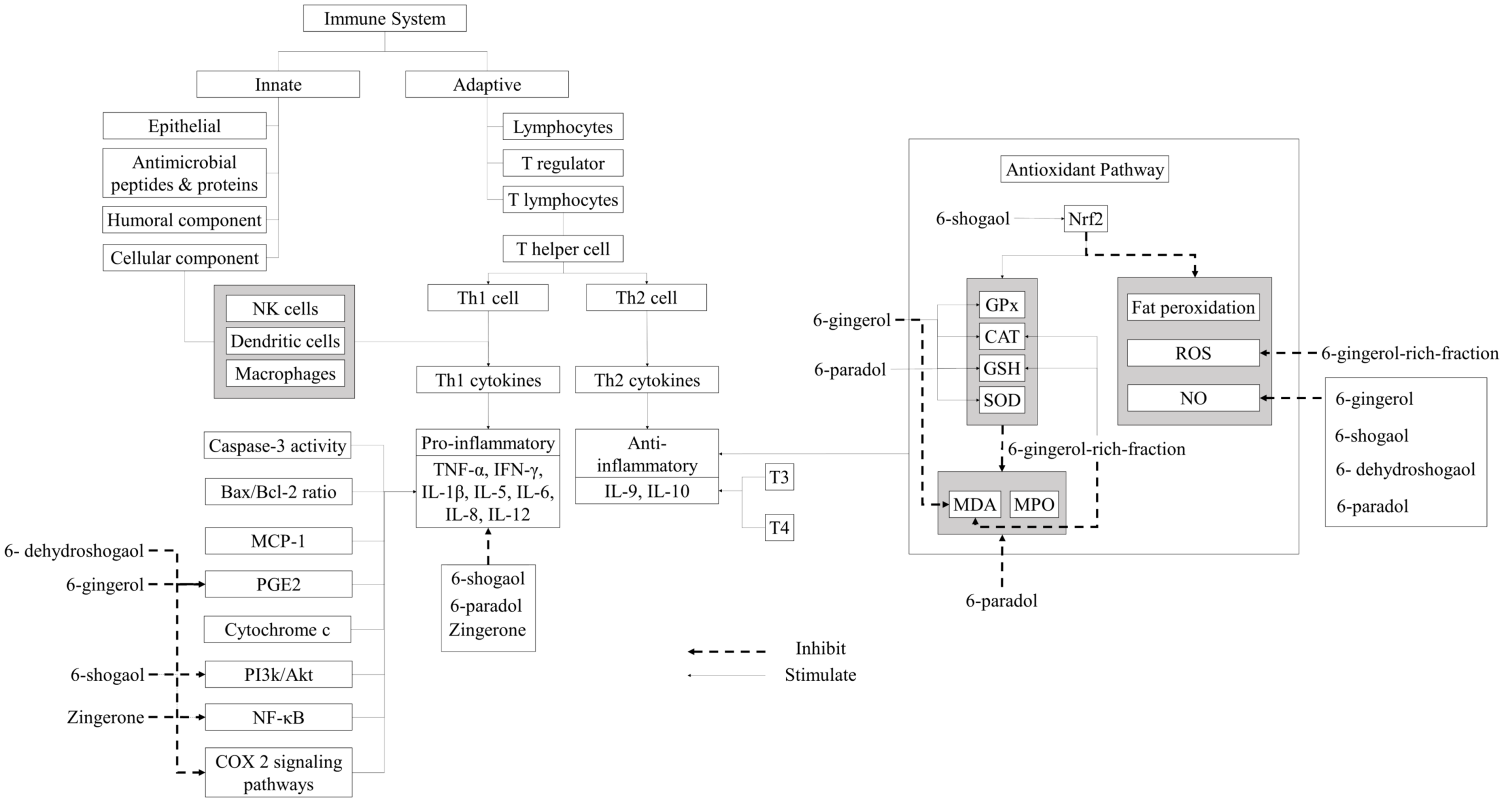
The potential of ginger as an immunomodulator.

Ginger’s antioxidant potential mechanism (Figure 4), related to bioactive components such as gingerol, shogaol, zingerone, and paradol. Inflammation is a defensive reaction of the body that arises after microbial invasion, exposure to antigens, or cellular and tissue injury. It entails intricate interactions among different cell types, mediators, receptors, and signaling pathways. Inflammation and oxidative stress can reciprocally influence each other, operating through the anti-inflammatory and antioxidant pathways. Immune cells, particularly T cells, govern the anti-inflammatory pathway. Th1 cell differentiation occurs subsequent to antigen recognition in the presence of IFNγ and IL-12. Th1 cells instigate cellular immunity, crucial for combatting intracellular pathogens. However, uncontrolled Th1 cell-related responses may provoke immunopathological reactions. In times of stress, the body naturally increases the number of Th1 cells. Elevated Th1 cell counts are observed in various diseases, such as multiple sclerosis. IL-12 also significantly contributes to Th1 cell differentiation. A robust correlation exists between IFNγ levels and enhanced activation of T lymphocytes, particularly CD4+ and CD8+ cells. IFNγ also promotes macrophage differentiation and regulates inducible nitric oxide synthase (iNOS) expression in dendritic cells and macrophages. Ginger modulates Th1 regulation by inhibiting IL-12 synthesis by macrophages, thereby affecting antigen presentation, T cell activation, and IFNγ and IL-12 secretion by T cells. Conversely, T lymphocytes transform into Th2 cells following antigen recognition in the presence of IL-4. Th2-specific transcription factors, like GATA 3 binding protein, stimulate Th2-related cytokine production (e.g., IL-4, IL-5, and IL-13). Th2 cells also induce B cells to generate immunoglobulins, fostering humoral immunity. Increased Th2 cell presence can curtail inflammation (55, 56).

Elevated levels of proinflammatory cytokines (e.g., IL-1β, IL-6, IL-23, IL-33, TNFα, and IL-17) and reduced levels of anti-inflammatory cytokines (such as IL-10, IL-27, and TGFβ) are commonly termed cytokine dysregulation. This imbalance is often observed in individuals facing stress conditions induced by free radicals or pathogenic infections. Dysregulation in cytokine production may contribute to the development of diseases associated with oxidative stress. Ginger is recognized for its anti-inflammatory properties, inhibiting cytokine formation linked with Th1 and Th2 cells and suppressing IgE production. Ginger modulates Th1-type cytokines (including IL-12, IFNγ, and TNF-α) and Th2-type cytokines (like IL-4 and IL-13), thereby reducing T lymphocyte activation and expansion. It also diminishes IFNγ production by T lymphocytes, suppresses IL-2 secretion by stimulated T lymphocytes, and interferes with IL-2 receptor signaling. Studies have shown ginger’s capacity to lower levels of IL-1, IL-6, and TNFα, as well as inflammatory markers like C-reactive protein. Ginger also inhibits NF-кB activation, decreases TNFα, IL-6, and IL-1β expression, and mitigates ROS production. Additionally, ginger suppresses the expression of iNOS, IL-6, and IL-8 through NF-κB inhibition (55, 56).

Reactive oxygen species (ROS) and reactive nitrogen species (RNS) are potent instigators of oxidative stress, provoking inflammatory responses. These radicals inflict damage upon essential biological molecules like lipids, proteins, DNA, and polysaccharides through peroxidation and nitrosation processes. This disturbance impairs normal cellular function and disrupts neuronal homeostatic equilibrium, ultimately culminating in apoptosis. Oxidative stress also impairs mitochondrial function, impeding adenosine triphosphate transport along the axon, potentially leading to neurodegeneration. Additionally, oxidative stress contributes to the escalation of inflammatory reactions via Nuclear Factor Light Chain-Enhancer (NF-κB) activation. Nitric oxide (NO) induces COX-2 expression, thereby elevating prostaglandin E2 synthesis. Cellular defenses against oxidative stress predominantly rely on the nuclear factor erythroid 2-related factor 2 (Nrf2), which orchestrates the expression of various antioxidant proteins and detoxification enzymes, including those involved in glutathione (GSH) synthesis, crucial for maintaining cellular homeostasis (55, 56).

Ginger displays antioxidant properties attributed to compounds like shogaol, gingerol, zingerone, and paradol, thereby diminishing ROS production. Ginger initiates the replenishment of antioxidant enzymes such as superoxide dismutase (SOD) and catalase (CAT), elevates glutathione levels, prevents lipid peroxidation, inhibits NO production, and neutralizes hydroxyl radicals. Furthermore, ginger notably suppresses iNOS expression, reduces the occurrence of caspase-3 positive cells, and diminishes TNF-α expression, thereby inhibiting ROS production and MAPK-related signaling. Additionally, it enhances the expression of specific antioxidant elements such as glutathione, heme oxygenase-1, and quinone-1 through Nrf2 activation. Moreover, ginger inhibits Bax apoptosis protein expression and prevents the expression of H202, malondialdehyde (MDA), and myeloperoxidase (MPO), also activated B cells’ phosphatidylinositol-3-kinase (PI3K) and protein kinase B (Akt), thus exerting a protective effect against cell damage induced by oxidative stress and inflammation (55, 56).

According to several studies on ginger antioxidants and anti-inflammatory (Table 4), ginger extract modulates the expression of Nrf2 target genes such as heme oxygenase-1 (HO-1), metallothionein 1 (MT1), aldo-keto reductase 1B10 (AKR1B10), ferritin light chain (FTL), and glutamyltransferase-like activity 4 (GGTLA4). This enhances the production of enzymatic antioxidants the same as SOD, CAT, and GPx, as well as the production of GSH. Increased antioxidant enzymes can reduce RONS synthesis as well as malondialdehyde (MDA) and myeloperoxide levels (MPO). Finally, ginger extract has been shown to reduce oxidative stress, lipid peroxidation, DNA damage, and cell death (43–51, 57).

Ginger tea is also known to suppress free radicals and inhibit fat peroxidation (54). Ginger oleoresin is also known to prevent cell damage by inhibiting ROS production, triggering Nrf2 translocation, and activating HO-1 and NQO1 gene expression (52). Studies related to the anti-inflammatory ability of ginger have shown that ginger extract can inhibit NF-ΚB and Akt thereby improving the expression of Cyclooxygenase-2 (COX-2) and iNOS in macrophage cells. Cyclooxygenase-2 (COX-2) is an enzyme that plays a role in the production of prostaglandin E2 (PGE2). Ginger extract can also reduce proinflammatory cytochrome IL-1β IL-6, TNF-α, cytochrome c, PGE2, and MCP-1. Ginger extract can inhibit the Bax/Bcl-2 ratio and caspase-3 activity and reduce cell damage by increasing T3 and T4. Ginger juice is known to inhibit NF-ΚB, thereby improving iNOS expression. Proinflammatory cytochromes such as TNF-, IL-6, and IL-1β are also inhibited to significantly reduce NO levels (53). NO works as a vasodilator that widens blood vessels so that the movement of immune cells to the site of infection becomes easier.

The body is constantly in an equilibrium state between free radical formation and elimination. Natural defense system against Catalase, glutathione reductase, SOD antioxidant enzymes, glutathione, urate, coenzyme Q, or exogenous stimuli such as bioactive substances like β-carotene, are all parts of the body’s natural defensive system against free radicals. Because of the neutrophil-produced free radicals, the immune system’s defense against infections serves as one of the sources of ROS generation. Micronutrient deficiency was known to cause a deterioration in immune function, both innate and adaptive, thereby rendering the body susceptible to pathogen attack as a consequence of immune imbalance. Thus, it was critical to consume bioactive compounds that serve as antioxidants in order to maintain a balanced immune response (58).

Ginger contains bioactive compounds, such as gingerol, shogaol, paradol, and zingerone. Those bioactive compounds were shown to have immunomodulatory properties due to their antioxidant, anti-inflammatory, and regulatory properties of innate and adaptive immune response components. These modulatory activities include inflammatory cytokines suppression and stimulation, antioxidative activity through ROS scavenging activity and enhancement of endogenous antioxidant defenses, and modulation of pro-inflammatory and anti-inflammatory signaling pathways. The potent bioactive components of ginger have been studied and shown that have antioxidant and anti-inflammatory activities as can be observed in Table 6. Some important components will be described in the next sub-sections.

**Table 6:** Ginger’s potential antioxidant and anti-inflammatory component.

Component	Study type	Subject	Dose	Results	Reference
6-gingerol	Pilot	Patients 18 years and over with a diagnosis of solid tumor	20 mg/day	Improves SOD, CAT, GPx and GSH. Lowers MDA and NOx	(59)
6-gingerol-rich-fraction	*In vivo*	Female wistar rat	50 mg/kg	Reduces H2O2 and MDA levels, increases antioxidant enzyme activity and GSH levels	(60)
6-gingerol-rich-fraction	*In vivo*	Male albino wistar rat	100 mg/kg	Reduces MDA levels, prevents depletion of calatase activity and GSH content	(61)
6-shogaol, 6-gingerol, 6- dehydroshogaol	*In vitro*	RAW 264.7 mouse macrophage cells	2.5, 5, 10 uM	Inhibits NO and PGE2 production	(62)
6-shogaol	*In vitro*	HaCaT cells	20 μM	Suppress UVB-induced inflammation, improve iNOS, COX-2, and TNF-α, and increase antioxidant enzymes through the Nrf/HO-1 pathway	(63)
6-shogaol	*In vitro*	Human intestinal epithelial cells HT-29/B6 and Caco-2	100 μM	Inhibits PI3k/Akt and NF-κB signaling pathways	(64)
6-paradol	*In vivo*	Sprague Dawley rat male	200 mg/kg	Improves GSH, MDA, MPO, IL-6, and TNF-α levels	(65)
6-paradol	*In vitro*	BV-2 microglia	20 μg/mL	Reduces NO, iNOS, IL-6, TNF-α production	(66)
	*In vivo*	Male ICR mice	10 mg/kg		
Zingerone	*In vivo*	BALB/c female mice	100 mg/kg	Inhibits NF-κB activity and lowers IL-1β levels	(51)
Zingerone	*In vitro*	MLE12 cells	50 mg/kg	Increases SOD, GSH, CAT, and IFN-γ activity. Decreased activity of MDA, IL-1β, IL-5, IL-13, TNF-α, NF-ΚB p65 expression	(67)

#### 3.4.1 Gingerol

Gingerol (23–35%) is a compound contained in ginger oleoresin which has a pale yellow color and is unstable (68). This gingerol gives a spicy taste to fresh ginger which is a phenolic compound (69). Gingerol is very susceptible to thermal decomposition (70). The compound 6-gingerol is reported to have antioxidant and anti-inflammatory properties (60, 71). The ability of gingerols, especially 6-gingerol, has been shown to increase SOD, CAT, GPx, and GSH levels, as well as reduce MDA and NOx in patients diagnosed with solid tumors aged 18 years and over. Ginger extract (20 mg/day) as a daily supplement for patients receiving chemotherapy can increase antioxidants compared to the placebo group. Ginger supplementation can increase antioxidant activity and reduce oxidative levels in patients (59). The 6-gingerol-rich fraction (50 mg/kg) can also reduce H_2_O_2_ and MDA levels, as well as increase antioxidant enzyme activity and rat GSH levels in *in vivo* studies (60). The ability of the 6-gingerol-rich fraction has also been known to reduce MDA levels and prevent depletion of catalase activity and GSH content in mice in 100 mg/kg dose (61). *In vitro* studies on mouse macrophages also demonstrated the ability of 6-gingerol to inhibit NO and PGE2 production (62).

#### 3.4.2 Shogaol

Shogaol (18–25%) is a constituent present in ginger, especially when ginger undergoes a drying or cooking process. The constituent shogaol may be made by the dehydrating reaction of gingerol, this causes the ginger to lose its pungency when cooked. Based on *in vitro* studies, 6-shogaol with 20 uM doses can suppress UVB-induced inflammation, improve iNOS, COX-2, and TNF-α, and increase antioxidant enzymes through the Nrf2 pathway in HaCaT cell culture, also can be a healing agent in cells undergoing oxidation so that it can prevent aging caused by free radicals (63). In human intestinal epithelial cells, 6-shogaol (100 uM) can also inhibit PI3k/Akt and NF-κB signaling pathways (64). 6-dehydroshogaol in 2.5 μM, 5 μM, and 10 μM doses the dehydrogenated form of shogaol is also known to have the ability to inhibit the production of NO and PGE2 in mouse macrophage cells (62).

#### 3.4.3 Paradol

Paradol is a derivative compound from gingerol and shogaol in ginger which has been known to have antioxidant and anti-inflammatory activity with no specific amount. 6-paradol can improve the levels of GSH, MDA, MPO, IL-6, and TNF-α by 200 mg/kg doses in mice (65). 6-paradol could reduce the production of NO, IL-6, and TNF-α in cultured BV-2 microglia cells by 20 ug/mL doses and prevent cell damage in mice by doses 10 mg/kg doses. Its mechanism is related to the inhibition of cyclooxygenase-2 enzyme production which modulates T cell immune response, including TNF-α as Th1 cytokines and IL-6 as Th2 cytokines. Paradol also known that have inhibition activity of iNOS upregulation which reduces NO, MDA, MPO production and increases GSH, so it can be prevent any cell damage (66).

#### 3.4.4 Zingerone

Zingerone is the primary flavor component of ginger which gives ginger its sweet taste when cooked. Fresh ginger does not contain zingerone, but is produced by cooking or drying ginger, which causes an inverse aldol reaction to gingerols with no specific amount (72). Zingerone proved to be a highly efficient free radical scavenger by inhibiting enzymes involved in the formation of RONS. *In vivo* studies show that zingerone can inhibit NF-ΚB activity and decrease IL-1β levels by 100 mg/kg doses in mice (51). In addition, it is also known that zingerone can increase the activity of SOD, GSH, CAT, and IFN-γ, and decrease the level of MDA, IL-1β, IL-4, IL-5, IL-13, TNF-, NF-ΚB p65 expression and p-IκB based on *in vitro* studies on MLE12 cell cultures by 50 mg/kg doses. Zingerone also support asthma therapy by decreasing NF-ΚB and activating the Nrf2/HO-1 signaling pathway through AMPK (67).

### 3.5 Ginger consumption as food and drink

Ginger’s potential as an antioxidant and anti-inflammatory will be effective only when consumed in adequate amounts. Ademosun, Omoba (73) mentioned that ginger was commonly consumed in Nigeria as a vegetable, spices, condiments, and drink. A commercial ginger drink containing 25 mg ginger root and other ingredients containing 6.89 mg GAE/L of total phenol and total flavonoid of 1.06 QE/L; on the other hand lab scale ginger drink containing ginger diluted with 200 mL water containing more than twice the total phenol, and more than five times the total flavonoids. Those bioactive compounds were responsible for generating a DPPH radical scavenging ability of about 30% from commercial drinks, and about 50% for 100% ginger drinks. Meanwhile, Naqvi, Thomson (74) used ginger powder to inject sous vide beef steak to 116% raw weight at 2 g/L ginger concentration to increase flavor. On the other hand Zagórska, Czernicka-Boś (75) review that ginger in home cooking has diverse final bioactivity depending on the cooking method used. Stewed ginger had TEAC (Trolox equivalent antioxidant capacity) of 19.3 μmol/g. meanwhile fried ginger without oil in a nonstick pan for 15 min producing ginger with IC_50_ of 950 μg/mL. The lack of agreement on the unit used to measure ginger antioxidant properties or integrated research that includes multiple ginger processing methods makes the interested parties have difficulties estimating the real benefit of consuming ginger with a certain processing method.

### 3.6 Challenges and future prospects of ginger

**Figure 5:**
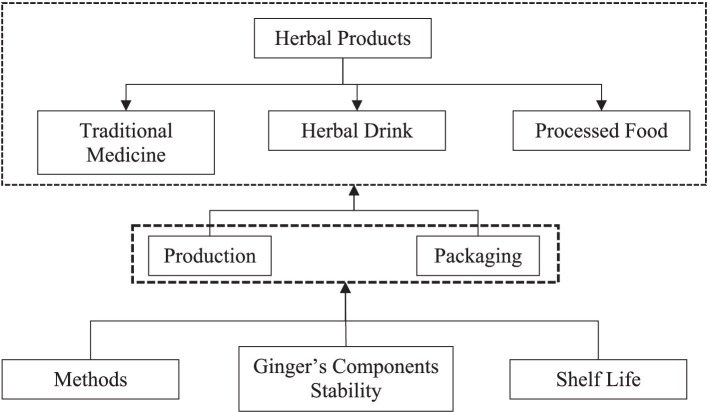
Challenges of ginger’s commercialization as herbal products.

There is always a potential for ginger to be commercialized as an herbal product (Figure 5). Nowadays, many people are selling herbal products in online shops, and it is increasing rapidly. Additionally, the herbal market is going to be high if the selection of products follows the trends, flows properly, and sells in the right time. The entrepreneurial spirit/thinking should go beyond anything other than the ability to mix or produce products. In Indonesia, herbal products based on ginger have been sold not only as traditional medicines or herbal drinks but also as processed food like candy and traditional cake. There should be consideration in having a proper and intensive education for developing ginger’s product in terms of production method, packaging, the stability of the ginger’s component, and shelf life of the herbal product. So, the products could be consumed, and the benefit of ginger is still contained in herbal product-processed food (76).

This review demonstrates that ginger contains various bioactive compounds that can be used as nutritional/therapeutic agents to modulate the immune system, inflammatory processes, antioxidant or redox balance, or immune components in general. To obtain a specific benefit from ginger, specific phytochemical compounds should be extracted at a particular condition to obtain an optimal yield (77).

The immune system’s ability to prevent diseases is strongly influenced by the host’s nutritional status. The discussion in this review suggested that ginger can be used as a potent immunomodulator and, when ingested, could enhance both cell-mediated and humoral immunity of the host, either through their antioxidant or anti-inflammation properties. Ginger has the capacity to enhance the pharmaceutical, food, medical, and agricultural industries. Ginger is becoming increasingly popular due to increased public health awareness due to its high nutritional content and health advantages.

Ginger-based nutraceuticals can be used as a supplement to help manage disease and maintain a healthy lifestyle. Other ginger-based products are also encouraged to be developed to create more diverse functional foods that can reach a broader consumer. Furthermore, ginger-based supplements can be developed as single compounds or phytocomplexes in the pharmaceutical industry. Ginger-based supplements or nutraceuticals can be used as human interventions as a complementary treatment in immune-related diseases after pre-clinical and clinical research.

Therefore, future collaborations between researchers from various fields, including chemists, biologists, clinicians, pharmacists, and the food industry, are required further to investigate the effect of ginger on human immunity. Collaboration between researchers and industry can help accelerate the advancement of ginger applications.

## 4 Conclusion

In conclusion, ginger has various bioactive components, such as gingerol, shogaol, zingerone, and paradol, known to have antioxidant and anti-inflammatory activities. Ginger extract, ginger juice, ginger tea, and ginger oleoresin have also been shown to have potential as immunomodulators through antioxidant and anti-inflammatory pathways. Bioactive compounds in ginger inhibit pro-inflammatory responses, increase levels of anti-inflammatory cytokines, and promote signaling pathways related to inflammation prevention on the anti-inflammatory pathway. Bioactive compounds in ginger are able to improve oxidative stress tolerance by eliminating ROS and lowering oxidative stress parameters, increasing antioxidant enzymes, and increasing antioxidant capacity.

Ginger’s ability to prevent oxidative stress and other diseases related to inflammation gives ginger the potential to be developed into various functional food products with education for developing ginger’s product. Furthermore, further research is needed regarding the toxicity of ginger and the daily consumption recommendation for humans.

## Author contributions

FA: Writing – review & editing, Writing – original draft, Project administration, Methodology, Funding acquisition, Conceptualization. GA: Writing – review & editing, Writing – original draft, Project administration, Funding acquisition, Conceptualization. AA: Writing – review & editing, Writing – original draft, Conceptualization. VF: Writing – review & editing, Writing – original draft, Supervision.
